# Sprint Acceleration Mechanical Outputs Derived from Position– or Velocity–Time Data: A Multi-System Comparison Study

**DOI:** 10.3390/s22228610

**Published:** 2022-11-08

**Authors:** Charly Fornasier-Santos, Axelle Arnould, Jérémy Jusseaume, Benjamin Millot, Gaël Guilhem, Antoine Couturier, Pierre Samozino, Jean Slawinski, Jean-Benoît Morin

**Affiliations:** 1 Laboratory Sport Expertise and Performance (EA 7370), French Institute of Sport (INSEP), 75012 Paris, France; 2 LAMHESS, University Côte d’Azur, 06000 Nice, France; 3 Laboratory of Metabolic Adaptations to Exercise under Physiological and Pathological Conditions (AME2P), University Clermont Auvergne (UCA), 63001 Clermont-Ferrand, France; 4 French Athletics Federation (FFA), 73376 Paris, France; 5 Interuniversity Laboratory of Human Movement Sciences, Univ Savoie Mont Blanc, EA 7424, 73000 Chambéry, France; 6 Inter-University Laboratory of Human Movement Biology, Univ Lyon, UJM-Saint-Etienne, EA 7424, 42023 Saint-Etienne, France

**Keywords:** GPS, linear encoder, force, velocity, sprint

## Abstract

To directly compare five commonly used on-field systems (motorized linear encoder, laser, radar, global positioning system, and timing gates) during sprint acceleration to (i) measure velocity–time data, (ii) compute the main associated force–velocity variables, and (iii) assess their respective inter-trial reliability. Eighteen participants performed three 40 m sprints, during which five systems were used to simultaneously and separately record the body center of the mass horizontal position or velocity over time. Horizontal force–velocity mechanical outputs for the two best trials were computed following an inverse dynamic model and based on an exponential fitting of the position- or velocity-time data. Between the five systems, the maximal running velocity was close (7.99 to 8.04 m.s^−1^), while the time constant showed larger differences (1.18 to 1.29 s). Concurrent validity results overall showed a relative systematic error of 0.86 to 2.28% for maximum and theoretically maximal velocity variables and 4.78 to 12.9% for early acceleration variables. The inter-trial reliability showed low coefficients of variation (all <5.74%), and was very close between all of the systems. All of the systems tested here can be considered relevant to measure the maximal velocity and compute the force–velocity mechanical outputs. Practitioners are advised to interpret the data obtained with either of these systems in light of these results.

## 1. Introduction

Sprint acceleration performance is key in many sports, and has been explored through the assessment of mechanical variables, leading more and more staff to assess them during sprinting (e.g., running velocity and ground reaction force). This has been done using gold standard 3D motion capture and force plates systems that measure the center of mass velocity of the sprinter [1,2,3]. However, analyzing a full acceleration phase of a sprint (from 0 to the achievement of maximal velocity in athletes) with such gold standard systems involves heavy experimental protocol and data processing procedures that may not consistently fit with in situ conditions and practitioners’ requirements. To solve this issue, a field method based on Newtonian laws of motion and athletes’ position or velocity measurement has been proposed and validated in order to compute the net step-averaged external force produced by athletes during a complete sprint acceleration [4,5]. This method has been used in various contexts of sports science and practice [6,7,8]. Provided the position–time or velocity–time input is accurately measured, this method can be theoretically implemented with a wide range of modern systems [4]. This accuracy of position–time or velocity–time measurement has been shown for linear encoder technology [9,10,11], lasers [12,13], radars [12,14], global positioning systems (GPS) [15,16], and timing gates [17]. On this basis, sprint specific horizontal force production capacities have recently been studied via the “force–velocity profile” approach using the 1080 Sprint motorized linear encoder technology [18], the MuscleLab laser system [19], Stalker ATS II radar [20], Catapult Vector S7 GPS [21], and the Microgate timing gates [22].

However, no study has directly assessed the concurrent validity of all these “silver standard” systems within a single protocol and a wide range of sprint velocities. Such a multiple-comparison study seems warranted to inform researchers and practitioners about the magnitude of inter-system differences and the potential interchangeability of the mechanical outcomes computed. The aim of this study was to directly compare five of the most commonly-used on-field systems (linear encoder, laser, radar, GPS, and timing gates) during sprint acceleration in order to (i) measure velocity–time data, (ii) compute the main associated force–velocity variables, and (iii) assess their respective inter-trial reliability.

## 2. Materials and Methods

### 2.1. Study Population

Eighteen participants (13 men and 5 women) (age: 27.0 ± 6.7 years, height: 1.79 ± 0.10 m, and body mass: 72.0 ± 12.6 kg) volunteered to participate in the present study. They were practicing sport at various levels ranging from non-specialist to elite sprinters, and were free from lower limb injuries in the three months prior to the tests. The participants were instructed to avoid any strenuous exercise 24 h prior to the experiments. They were informed about the nature, aims, and risks associated with the experimental procedures before providing written consent to participate in the present study, which was approved by the ethical committee of the French west area, Tours (N°2021-A02523-38).

### 2.2. Protocol

After an individual 20 min warm-up (mobility, activation, athletics drills, and accelerations), each participant performed three 40 m sprint trials on an official outdoor track (tartan^®^), with a three-point start. The following systems were used synchronously to measure the horizontal velocity or displacement of the body center of mass during the trials: (i) linear motorized encoder (1080 Sprint, 1080 Motion; 333 Hz, Lidingö, Sweden), (ii) laser (Muscle Lab^TM^ Laser Speed device Ergotest Innovations, Stathelle, Norway, 1000 Hz), (iii) radar (Stalker Pro II Sports Radar Gun; Plano, TX, USA, 46.875 Hz), (iv) GPS (Vector S7; Catapult Innovations, Melbourne, Australia, 10 Hz), and (v) timing gates (Witty Microgate, Microgate, Bolzano, Italy). The two best 40 m sprints based on the fastest finish time at 40 m were retained for the analysis. For each trial and each system, position–time and velocity–time data were used to compute the sprint mechanical outputs using the same data analysis procedure (as detailed below) and compared. The following sections present each system’s characteristics and the associated raw data recording. All systems simultaneously and separately captured running speed, but were not time-synchronized due to obvious technical limitations.

#### 2.2.1. Linear Encoder

The device was set in isotonic mode with 1 kg of resistance load in order to reduce the slack of the cable, and the device was placed on the track, 5 m behind the starting position. However, this extra 1 kg of resistance did not affect the inter-system comparisons for running velocity and derived data, as all systems measured the same running movement with the runner connected to the encoder via a cable attached to a waist belt. Raw velocity data were computed at 333 Hz from the change in position of the cable and were recorded on a tablet. Raw velocity data were exported in ASCII format for further analysis with OriginPro, version 2021 (OriginLab Corporation, Northampton, MA, USA).

#### 2.2.2. Laser

The device was set on a tripod on the track, 5 m behind the starting position and 1 m above ground level, corresponding approximately to the height of participants’ centre of mass [14]. Laser system calculate velocity measuring the time delay of pulsed infrared light that is reflected off the subject [12]. Raw velocity data were sampled at 1000 Hz, recorded and smoothed by the manufacturer software (Muscle Lab^TM^, version 10.200.90.5097, Stathelle, Norway), and then exported in ASCII format for further analysis with OriginPro.

#### 2.2.3. Radar

The device was set on a tripod on the track, 5 m behind the starting position and 1 m above ground level. The raw data sampled participants’ running velocity from very high frequency radio waves converted into a stream of digital data processed with a custom made software (MookyStalker V2.0.9) to provide the velocity at a sampling frequency of 46.875 Hz [12]. Raw data were then exported in TXT format for further analysis with OriginPro. Raw data outliers were deleted. Then, the cleaned data were fitted using the exponential model proposed and were validated by Samozino and colleagues [5] in order to compute the sprint mechanical outputs.

#### 2.2.4. GPS

The GPS units provided a sampling rate of 10 Hz and encompassed a double constellation system (GNSS and GPS). They were tightly installed into a fitted vest on the upper thoracic spine between the scapulae. Each participant carried two GPS devices within two different vests at the same time to examine the inter-device variability. The GPS positioning quality was 61.9 ± 5.3%, the average horizontal dilution of precision was 0.65 ± 0.04, and the number of satellites was 17.0 ± 1.0, which is considered to be within the upper range of good signal quality [23]. GPS can calculate the velocity using either positional differentiation or Doppler-shift methods. The GPS brand used in the present study determined the velocity via the Doppler-shift method, which is based on the velocity computation by measuring the change in frequency of the satellite emitted periodic signal [23]. The raw velocity data recorded by each GPS unit were uploaded to the Openfield console (version 3.4.0, Catapult Innovations, Melbourne, Australia) with the CSV format box checked, allowing for access to the both raw and smoothed velocity data. Finally, data were analyzed with OriginPro.

#### 2.2.5. Timing Gates

Dual-beam timing gates were placed on the track 1 m above ground level at 0, 5, 10, 15, 20, 30, and 40 m from the starting line, to capture the entire acceleration phase and ensure valid sprint mechanical values computation [17]. The starting position was located 0.5 m behind the first timing gate (i.e., 0 m). As a result of this location, the time delay between the first propulsive action of the participant (i.e., determined when the thumb of the forward hand took off the ground) and the crossing of the timing gates was determined frame by frame by visual inspection from the video recordings using an iPhone 8 (iOS 13.7, Apple Inc., Los Altos, CA, USA). Videos were recorded at 240 fps from a left lateral view located 5 m from the start line. The time delay was computed on 36 sprint trials and its value was 0.25 ± 0.06 s.

### 2.3. Data Processing

For all of the systems, the raw position–time or velocity–time data for the two best trials were used to compute the mechanical outputs based on the method proposed by Samozino and colleagues [4,5]. Briefly, in this macroscopic method based on the motion of the body center of mass, running position–time or velocity–time over time during the acceleration was fitted with an exponential function using the least-square regression method with a time adjustment to ensure the actual start of the computation at t = 0 s, in case of delay between the time trigger and the actual increase in velocity [4] with V_max_, the maximal velocity, and Tau, the early acceleration time-constant:

$$v\left(t\right)=v_{\max}\cdot \left(1-e^{-\left(t-d\right)/\tau }\right)$$

**Figure 1:**
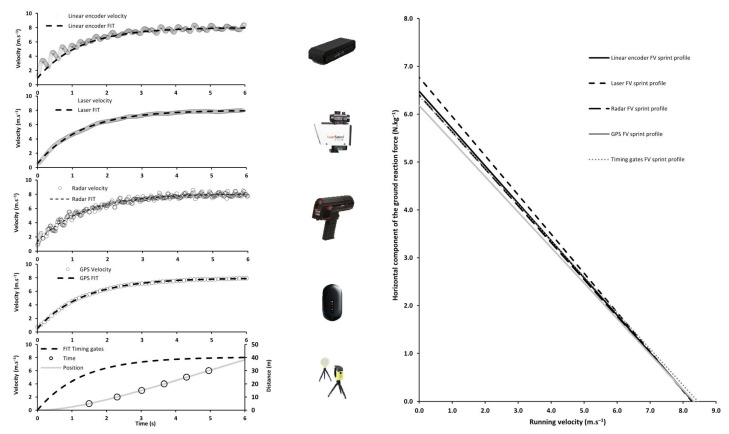
Raw velocity data and raw velocity data fitted by the mono-exponential function [5], and force–velocity profiles for the two best trials were subsequently computed for each system (linear encoder, laser, radar, GPS, and timing gates). The maximal velocity (V_max_), acceleration time constant (Tau), theoretical maximal velocity (V_0_), and horizontal component of the ground reaction force (F_0_) were calculated.

Then, the acceleration of the runner in the horizontal direction was computed by derivating velocity over time, and following the fundamental laws of dynamics in the horizontal direction, the net horizontal antero–posterior ground reaction force applied to the body center of mass was modelled over time based on the athlete’s body mass and the estimated aerodynamic drag force. Finally, the linear force–velocity relationship obtained was described by theoretical maximal velocity (V_0_) and the horizontal component of the ground reaction force (F_0_) (Figure 1). For the velocity recording systems, data considered for further analysis ranged between the first value over 0.5 m.s^−1^ and the last velocity point of the V_max_ plateau.

### 2.4. Statistics

**Figure 2:**
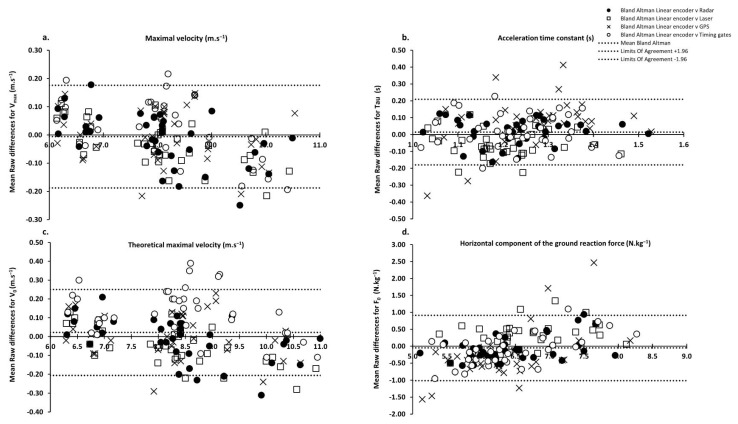
Bland and Altman comparisons between the linear encoder and other systems [24]. Mean raw differences with the linear encoder were computed for (**a**) maximal velocity (V_max_), (**b**) acceleration time constant (Tau), (**c**) theoretical maximal velocity (V_0_), and (**d**) horizontal component of the ground reaction force (F_0_).

All data are presented as mean ± standard deviation (SD) (min–max). The main kinetic parameters of the force–velocity relationship obtained with each system were compared using bias (mean differences between systems and systematic bias) and limits of agreement (bias + or—random errors computed as 1.96 × standard deviation of the inter-system differences [24] (Figure 2). The inter-trial reliability for each parameter was calculated using the change in the mean, and the standard error of measurement (SEM, expressed in percentage of mean values) between the two best trials [25]. In addition, this inter-trial coefficient of variation (%) was computed for the main variables.

## 3. Results

### 3.1. Inter-System Comparisons

Table 1 shows the main sprint variables computed for all of the participants and trials (n = 36) on a 40 m sprint for the five considered systems. These results showed almost equal values between systems for V_max_ and V_0_ and especially between the GPS and linear encoder. However, greater differences occurred between the systems for Tau and F_0_, with extreme differences observed between GPS and the linear encoder. In addition, we tested two GPS units simultaneously, but for clarity reasons, we only presented the results of unit 1, due to the close results (less than 0.2% for V_0_ and 2.2% for F_0_) observed between unit 1 and unit 2.

Table 2a,b presents the mean raw differences between the systems and the associated limits of agreement for all of the subjects and trials.

### 3.2. Inter-Trial Comparisons

Table 3 shows the inter-trial coefficient of variation mean (CV) and the associated changes in the mean and standard error of measurement (SEM) for all of the subjects and trials (n = 36) using the five systems compared. These results overall show similar CV and SEM for V_max_, Tau, V_0_, and F_0_ between the trials.

## 4. Discussion

The aim of this study was to compare five of the most popular field systems (linear encoder, laser, radar, GPS, and timing gates) during sprint acceleration to (i) measure velocity–time data, (ii) compute the main associated force–velocity variables, and (iii) assess their respective inter-trial reliability.

The present findings confirm the almost perfect goodness of fit between raw velocity or position data and the exponential model, and in turn the feasibility of the force–velocity relationship determination following the method proposed and validated by Samozino and colleagues [4,5], based on earlier observations [9,26,27]. This feasibility is confirmed for all of the systems tested, as shown in previous studies [18,19,20,21,22]. Concurrent validity results overall show a small relative systematic error of 0.85% to 2.28% for the maximum velocity variables (V_max_ and V_0_) and larger (4.78% to 12.9%) for the early acceleration variables (Tau and F_0_).

### 4.1. Inter-System Comparisons

In the absence of an absolute gold standard, the concurrent validity results for V_max_ show a relative systematic error ranging from 0.86 ± 0.47% between the radar and laser to 1.06 ± 0.77% between the GPS and laser. As also shown in Figure 1 and in Table 2b, these results for V_0_ show the same trend with slightly larger ranges of mean raw differences: from 1.06 ± 0.87% between the radar and linear encoder to 2.28 ± 1.49% between the timing gates and laser. This suggests that all of the systems tested could be considered interchangeable to assess V_max_ and V_0_. This result is in accordance with the findings of some previous studies comparing some of these systems [12,21,28,29,30]. This very high level of agreement was probably due to the low rate of change in the running velocity around the V_max_ plateau.

Regarding the early acceleration variables (Tau and F_0_), all comparisons showed greater inter-system differences: relative systematic error for Tau ranging from 5.49 ± 3.72% between the radar and linear encoder to 12.89 ± 9.09% between the laser and GPS. For F_0_, they ranged from 4.78 ± 3.20% between the linear encoder and radar to 11.54 ± 6.29% between the laser and GPS. As the rate of velocity increase was higher in the early acceleration phase, this likely exacerbated the difference found between systems, compared to the lower rate of change in velocity by the end of the acceleration. Contrary to the maximum velocity variables, these results confirm the reliability of intra-system comparisons and suggest that comparisons of athlete’s values obtained from different systems should take these differences into account.

Despite consistent overall results, the data from Table 1 show a specific difference between timing gates (8.45 m.s^−1^ on average) and all others systems (from 8.27 to 8.33 m.s^−1^ on average) for V_0_. This could be due to the 40 m split time measurements for all trials, while the velocity data systems have been analyzed up to the last point of the V_max_ plateau, which occurred before 40 m for most of the athletes tested. The present sample of participants did not include world-class level sprinters, but data were consistent across the wide range of V_max_ tested (6.5 to 10.60 m.s^−1^).

The inter-system differences observed for Tau and F_0_ may be explained by the fitting method used in this experimentation, and the first data taken into account after the velocity threshold of 0.5 m.s^−1^, which could have a major influence on the model. In line with the interpretation of the good agreement for the maximal velocity values, the lower agreement for acceleration variables could also be due to the very high rate of change in velocity during early acceleration. This influence was even higher when the sample rate of the system was lower.

Regarding the laser and GPS systems, the set of data analyzed was the smoothed data provided by the manufacturer software, but we also analyzed the complete set of raw data (specifically provided by the manufacturer for the purpose of this study). In both cases, the results for the raw data were extremely close to those presented in the present study for the smoothed data, which are the only data accessible by default to all users. Indeed, less than 1% difference was observed on the relative systematic error values between the raw and smoothed data for all of the systems and variables.

### 4.2. Inter-Trial Comparisons

The inter-trial reliability showed low to very low coefficients of variation (highest CV of 5.74%) and very close results between all of the systems (Table 3). These results showed that the inter-trial reliability was good in the population tested, and that each of the five field systems tested could be considered equally reliable for intra- or inter-athlete comparisons using the same system, measurement, and analysis procedures.

### 4.3. Limitations

All of the systems used did not measure the velocity of the center of the mass and thus induced uncertainty regarding the real velocity of the participants. Moreover, this study did not include a gold standard (3D camera or force platform), because it was a very rare setting, and was almost not possible to use in such field conditions. In this context, we focused on the comparisons of systems considered as “silver standards”.

Split times obtained with slow motion video analysis (MySprint app or GoPro camera) [22,31] is another popular field method that has not been tested here. This was due to logistical reasons in a heavy protocol context, but we can reasonably assume that the results of this split times approach would have been very close to other split times systems obtained here, as presented in previous studies [22,31].

We cannot rule out the influence of the fixed starting threshold at 0.5 m.s^−1^ and the visual inspection of the V_max_ plateau on the computation outcomes. Ongoing research has been undertaken to test this hypothesis. However, the same methodological approach based on signal visual inspection has been used by the same experienced investigator (author CFS) for each technology.

Finally, 1 kg of resistance was used on the motorized linear encoder for each trial to prevent any form of slack in the cable, but this did not affect the inter-system comparisons performed in our study. That being said, this information needs to be taken into account when comparing the present data to other studies using other systems that do not include this slight extra resistance.

The number of satellites and the average horizontal dilution of precision are key indicators to ensure that the data collected can be analyzed and interpreted with confidence. Indeed, poor quality data (e.g., less than 6 satellites connected to the devices and/or horizontal dilution of precision greater than 1) [23] could give erroneous values of speed and acceleration measures that can influence the interpretation of the sprint mechanical variables computed. Therefore, practitioners should systematically take into account these indicators to analyze the data with confidence.

## 5. Conclusions

Given the inter-system and inter-trial differences observed, all of the systems tested here can be considered relevant to measure the maximal velocity and compute the force–velocity sprint profile outputs using Samozino et al.’s validated approach. However, researchers and practitioners are advised to interpret their own data obtained with either of these systems in light of these results, and their cost/ease-of-use ratio.

Only intra-system comparisons will allow for the most accurate interpretation of inter-athletes or intra-athlete changes. Alternatively, inter-system comparisons (for example between different research studies) must be interpreted cautiously due to the percent differences observed here and the different data sampling and processes. Some of our recent unpublished works show that higher speed thresholds to determine the sprint start (e.g., 1 m.s^−1^) lead to higher inter-systems reliability.

These results suggest the possibility of several practical applications such as facilitated monitoring and regular analysis of the sprint mechanical profile in prevention or injury management, on the basis of several different systems.

## Figures and Tables

**Table 1:** Main mechanical variables computed from position or velocity data collected during 40 m sprints for all participants and trials (n = 36) using the five systems compared. Values are mean (in bold) ± SD, (min–max). Numbers in italic indicate the inter-subjects’ coefficient of variation (%) for maximal velocity (V_max_), acceleration time constant (Tau), and theoretical maxima of the force–velocity relationship (F_0_ and V_0_).

	V_max_ (m.s^−1^)	Tau (s)	V_0_ (m.s^−1^)	F_0_ (N.kg^−1^)
**Linear encoder**	**8.02** ± 1.20 (6.11–10.50) *15.0%*	**1.22** ± 0.12 (1.00–1.52) *9.4%*	**8.31** ± 1.30 (6.26–11.00) *15.7%*	**6.48** ± 0.62 (5.21–8.09) *9.6%*
**Laser**	**7.99** ± 1.15 (6.17–10.37) *14.4%*	**1.17** ± 0.11 (0.99–1.40) *9.0%*	**8.27** ± 1.24 (6.33–10.83) *15.0%*	**6.77** ± 0.77 (5.30–8.15) *11.4%*
**Radar**	**8.00** ± 1.15 (6.17–10.49) *14.4%*	**1.24** ± 0.12 (1.03–1.52) *9.4%*	**8.30** ± 1.24 (6.31–10.99) *15.0%*	**6.40** ± 0.78 (5.01–8.00) *12.3%*
**GPS**	**8.02** ± 1.18 (6.14–10.57) *14.7%*	**1.29** ± 0.16 (0.85–1.55) *12.3%*	**8.33** ± 1.28 (6.28–11.09) *15.4%*	**6.18** ± 0.96 (4.36–8.88) *15.6%*
**Timing gates**	**8.04** ± 1.15 (6.16–10.30) *14.3%*	**1.25** ± 0.11 (0.98–1.45) *8.9%*	**8.45** ± 1.27 (6.39–10.89) *15.0%*	**6.38** ± 0.87 (4.85–8.45) *13.7%*

**Table 2:** (**a**). Mean raw differences (in bold) for two by two comparisons (line vs. column = one comparison) ± SD and associated limits of agreement for all subjects and trials (n = 36) for maximal velocity (V_max_, lower part of the table, background in grey), acceleration time constant (Tau, upper part, background in white). (**b**). Mean raw differences (in bold) for two by two comparisons (line vs. column = one comparison) ± SD and associated limits of agreement for all subjects and trials (n = 36) for theoretical maximal velocity (V_0_, lower part of the table, background in grey) force and velocity (F_0_, upper part, background in white).

(a)						
	Linear Encoder	Laser	Radar	GPS	Timing Gates	
**Linear encoder**		**−0.055** ± 0.079 (−0.210; 0.100)	**0.018** ± 0.077 (−0.133; 0.169)	**0.072** ± 0.140 (−0.203; 0.346)	**0.025** ± 0.101 (−0.147; 0.223)	**Tau (s)**
**Laser**	**−0.031** ± 0.080 (−0.187; 0.125)		**−0.072** ± 0.077 (−0.224; 0.079)	**0.126** ± 0.135 (−0.139; 0.391)	**0.079** ± 0.095 (−0.108; 0.267)	
**Radar**	**−0.014** ± 0.093 (−0.196; 0.167)	**−0.017** ± 0.078 (−0.169; 0.136)		**0.054** ± 0.148 (−0.235; 0.343)	**0.007** ± 0.096 (−0.181; 0.195)	
**GPS**	**−0.003** ± 0.096 (−0.191; 0.186)	**0.028** ± 0.104 (−0.177; 0.233)	**0.012** ± 0.100 (−0.184; 0.207)		**−0.047** ± 0.167 (−0.374; 0.280)	
**Timing gates**	**0.025** ± 0.103 (−0.177; 0.226)	**0.056** ± 0.097 (−0.135; 0.246)	**0.039** ± 0.100 (−0.156; 0.234)	**0.027** ± 0.107 (−0.182; 0.237)		
	**V_max_ (m.s^−1^)**					
**(b)**						
	**Linear Encoder**	**Laser**	**Radar**	**GPS**	**Timing Gates**	
**Linear encoder**		**0.294** ± 0.397 (−0.485; 1.072)	**−0.086** ± 0.375 (−0.820; 0.649)	**−0.299** ± 0.729 (−1.729; 1.131)	**−0.104** ± 0.462 (−1.009; 0.800)	**F_0_ (N.kg^−1^)**
**Laser**	**−0.047** ± 0.102 (−0.246; 0.153)		**0.379** ± 0.393 (−0.391; 1.150)	**−0.593** ± 0.677 (−1.919; 0.733)	**−0.398** ± 0.475 (−1.329; 0.533)	
**Radar**	**−0.013** ± 0.116 (−0.239; 0.214)	**−0.034** ± 0.099 (−0.228; 0.160)		**−0.214** ± 0.791 (−1.764; 1.337)	**−0.019** ± 0.460 (−0.920; 0.882)	
**GPS**	**0.012** ± 0.122 (−0.227; 0.252)	**0.059** ± 0.135 (−0.206; 0.323)	**0.025** ± 0.132 (−0.234; 0.284)		**0.195** ± 0.857 (−1.484; 1.874)	
**Timing gates**	**0.137** ± 0.126 (−0.110; 0.384)	**0.183** ± 0.133 (−0.078; 0.445)	**0.149** ± 0.134 (−0.114; 0.413)	**0.124** ± 0.141 (−0.152; 0.400)		
	**V_0_ (m.s^−1^)**					

**Table 3:** The inter-trial coefficient of variation mean in bold (CV in %) and associated change in the mean (bold) and standard error of measurement (SEM) for all subjects and trials (n = 36) for the five systems compared. Relative change in the mean and relative SEM in % and italic.

	CV Inter Trial Mean ± SD (%)				Change in the Mean ± SEM			
	V_max_ (%)	Tau (%)	V_0_ (%)	F_0_ (%)	V_max_ (m.s^−1^)	Tau (s)	V_0_ (m.s^−1^)	F_0_ (N.kg^−1^)
**Linear encoder**	**1.13** ± 0.98	**4.52** ± 3.04	**1.25** ± 1.13	**3.84** ± 2.85	**0.04** ± 0.13 *0.5 ± 1.6%*	**−0.04** ± 0.06 *−3.0 ± 5.2%*	**0.04** ± 0.16 *0.5 ± 1.9%*	**0.21** ± 0.27 *3.3 ± 4.2%*
**Laser smooth**	**1.11** ± 0.77	**4.58** ± 3.33	**1.26** ± 0.79	**4.11** ± 3.37	**0.05** ± 0.11 *0.6 ± 1.4%*	**−0.03** ± 0.06 *−2.6 ± 5.5%*	**0.05** ± 0.13 *0.6 ± 1.6%*	**0.20** ± 0.33 *3.0 ± 4.9%*
**Radar**	**1.37** ± 0.94	**5.00** ± 4.27	**1.52** ± 1.06	**4.32** ± 3.93	**0.08** ± 0.13 *1.0 ± 1.7%*	**−0.01** ± 0.08 *−0.9 ± 6.5%*	**0.09** ± 0.16 *1.0 ± 1.9%*	**0.09** ± 0.39 *1.4 ± 6.1%*
**GPS smooth**	**1.47** ± 1.40	**5.74** ± 3.98	**1.63** ± 1.57	**5.64** ± 5.89	**0.07** ± 0.18 *0.9 ± 2.2%*	**0.03** ± 0.09 *2.0 ± 6.7%*	**0.08** ± 0.20 *1.0 ± 2.5%*	**−0.06** ± 0.40 *−1.1 ± 6.4%*
**Timing gates**	**1.31** ± 1.00	**5.09** ± 2.06	**1.47** ± 1.12	**4.20** ± 2.68	**0.04** ± 0.14 *0.5 ± 1.7%*	**−0.04** ± 0.06 *−3.5 ± 4.9%*	**0.03** ± 0.16 *0.4 ± 1.9%*	**0.23** ± 0.29 *3.6 ± 4.6%*

## Data Availability

Not applicable.
